# The effects of three quarter and full length foot orthoses on patellofemoral pain sufferers when walking and descending stairs

**DOI:** 10.1186/1757-1146-7-S1-A70

**Published:** 2014-04-08

**Authors:** Jim Richards, John Burston, James Selfe

**Affiliations:** 1 Allied Health Professions Research Unit, University of Central Lancashire, Preston, PR1 2HE, UK

## Background

Patellofemoral pain is a common disorder whose aetiology is multifactorial and is often attributed to foot function. Foot orthoses are commonly prescribed for this condition; however the mechanisms by which they work are poorly understood. Previous studies using single segment foot models have hypothesised that it may be control of the midfoot which holds the key to understanding orthotic control. Over the last decade it has become possible to divide the foot into multiple segments, however little work exists investigating the use of orthoses on different segments of the foot in this patient group. The aim of this study was to investigate the differences in the kinematics and kinetics of the lower limb during walking and step descent between patellofemoral patients and normal subjects and the effect of ¾ and full length foot orthoses versus no intervention.

## Method

Kinematic and kinetic data were recorded from 15 healthy subjects and 15 patients diagnosed with patellofemoral pain using 10 Oqus cameras and 4 AMTI force platforms. Subjects were asked to walk at a self-selected pace and complete a 20cm step down. The foot was modelled using a three segment 6 degrees of freedom model by fixing the marker set directly to the shoes and the lower limb was modelled using the calibrated anatomical systems technique.

## Results

Significant differences were seen between the healthy subjects and the patellofemoral pain patients during both tasks at the midfoot and rearfoot movement in the sagittal and coronal planes (p=0.003 to 0.016); at the knee joint significant differences were seen in the sagittal, coronal and transverse plane movement (p=0.001 to 0.01); and in the moments about the ankle and knee joints in the sagittal and coronal planes (p=0.012 to 0.035). The orthoses produced statistically significant differences in the movement in the forefoot, midfoot and rearfoot across all three planes for both tasks (p=0.001 to 0.032). The orthoses showed no change in the knee kinematics, although a significant reduction in the knee coronal plane moments during step descent was seen in both the ¾ and full length foot orthoses (p=0.019, p=0.028).

## Conclusions

Despite placing markers on the shoes this study was able to detect significant differences within the foot segments and identified potentially clinically important differences between patellofemoral pain patients and normal subjects and was able to determine clinical important changes due to treatment.

